# Thriving in the Cold: Glacial Expansion and Post-Glacial Contraction of a Temperate Terrestrial Salamander (*Plethodon serratus*)

**DOI:** 10.1371/journal.pone.0130131

**Published:** 2015-07-01

**Authors:** Catherine E. Newman, Christopher C. Austin

**Affiliations:** 1 Museum of Natural Science, Louisiana State University, Baton Rouge, Louisiana, United States of America; 2 Department of Biological Sciences, Louisiana State University, Baton Rouge, Louisiana, United States of America; University of Colorado, UNITED STATES

## Abstract

The dynamic geologic history of the southeastern United States has played a major role in shaping the geographic distributions of amphibians in the region. In the phylogeographic literature, the predominant pattern of distribution shifts through time of temperate species is one of contraction during glacial maxima and persistence in refugia. However, the diverse biology and ecology of amphibian species suggest that a “one-size-fits-all” model may be inappropriate. Nearly 10% of amphibian species in the region have a current distribution comprised of multiple disjunct, restricted areas that resemble the shape of Pleistocene refugia identified for other temperate taxa in the literature. Here, we apply genetics and spatially explicit climate analyses to test the hypothesis that the disjunct regions of these species ranges are climatic refugia for species that were more broadly distributed during glacial maxima. We use the salamander *Plethodon serratus* as a model, as its range consists of four disjunct regions in the Southeast. Phylogenetic results show that *P*. *serratus* is comprised of multiple genetic lineages, and the four regions are not reciprocally monophyletic. The Appalachian salamanders form a clade sister to all other *P*. *serratus*. Niche and paleodistribution modeling results suggest that *P*. *serratus* expanded from the Appalachians during the cooler Last Glacial Maximum and has since been restricted to its current disjunct distribution by a warming climate. These data reject the universal applicability of the glacial contraction model to temperate taxa and reiterate the importance of considering the natural history of individual species.

## Introduction

The southeastern United States has a rich geologic and biogeographic history [1–3] and contains significant spatial clustering of phylogenetic breaks for trees, birds, and mammals [4], reptiles [5,6], and amphibians [7]. Amphibian species in this region show a wide variety of distribution patterns, including, for example, widespread ranges (e.g., *Rana sphenocephala*), disjunct distributions (e.g., *Hyla andersonii*), and very small ranges encompassing only a single mountaintop or cave (e.g., *Gyrinophilus subterraneus*). Phylogeographic research tends to focus on either end of the spectrum due to unique qualities of these taxa: widely distributed species often contain multiple cryptic lineages, and species with extremely small ranges are often of conservation concern. But little is known about the phylogeographic history of species with distributions that are both restricted and disjunct. Eighteen of 144 amphibian species in the Southeast [8,9] have a geographic distribution consisting of at least three disjunct regions, but none of these species has been the focus of intensive phylogeographic study. Advances in molecular methods and the wide availability of specimen collection data and climate layers have facilitated studies integrating phylogenetics and spatially-explicit climate and niche analyses. For the first time, we apply these methods to a southeastern amphibian species with a disjunct distribution to investigate its evolutionary history and explore broader questions about amphibian phylogeographic patterns in this region.

Disjunct species distributions provide an intriguing backdrop for phylogeographic studies because they reflect some level of isolation among populations, which directly affects patterns of genetic variation [10]. One possible explanation for these distributions is that the disjunct regions are refugial areas for species that were once more broadly distributed and have been restricted by a warming climate since the Last Glacial Maximum (LGM) [11]. However, this response is usually associated with alpine and other cold-adapted species and is contrary to the pattern commonly cited in the literature for temperate species, which often describe post-glacial expansion from refugia [12]. Often, a species range is inferred to have contracted during the LGM, as ice cover and unsuitably cold and dry climates forced species into glacial refugia, from which they subsequently expanded as the climate warmed [3,4]. But despite the historical focus on locating glacial refugia, it has become apparent that this model of glacial contraction is not universally applicable, even to systems for which it might typically be assumed. For example, arid-adapted biota of Australia were shown to fit a model of glacial expansion, contrary to the common assumption of contraction to refugia [13]. In addition, a recent study of the European temperate frog species *Hyla sarda* demonstrated that the lower sea levels during the LGM may have created new available suitable habitat for that species, enabling range expansion [14].

Terrestrial salamanders of the genus *Plethodon* are unique among southeastern amphibians in that they do not require creeks or vernal pools for reproduction or larval development. Rather, these salamanders are direct developing and require only sufficient moisture for eggs and adult cutaneous respiration. It is possible, then, that some of these species, especially the ones currently found at higher elevations and cooler climates, flourished during the drier and cooler glacial maxima, rather than contracting into refugial areas.

**Fig 1:**
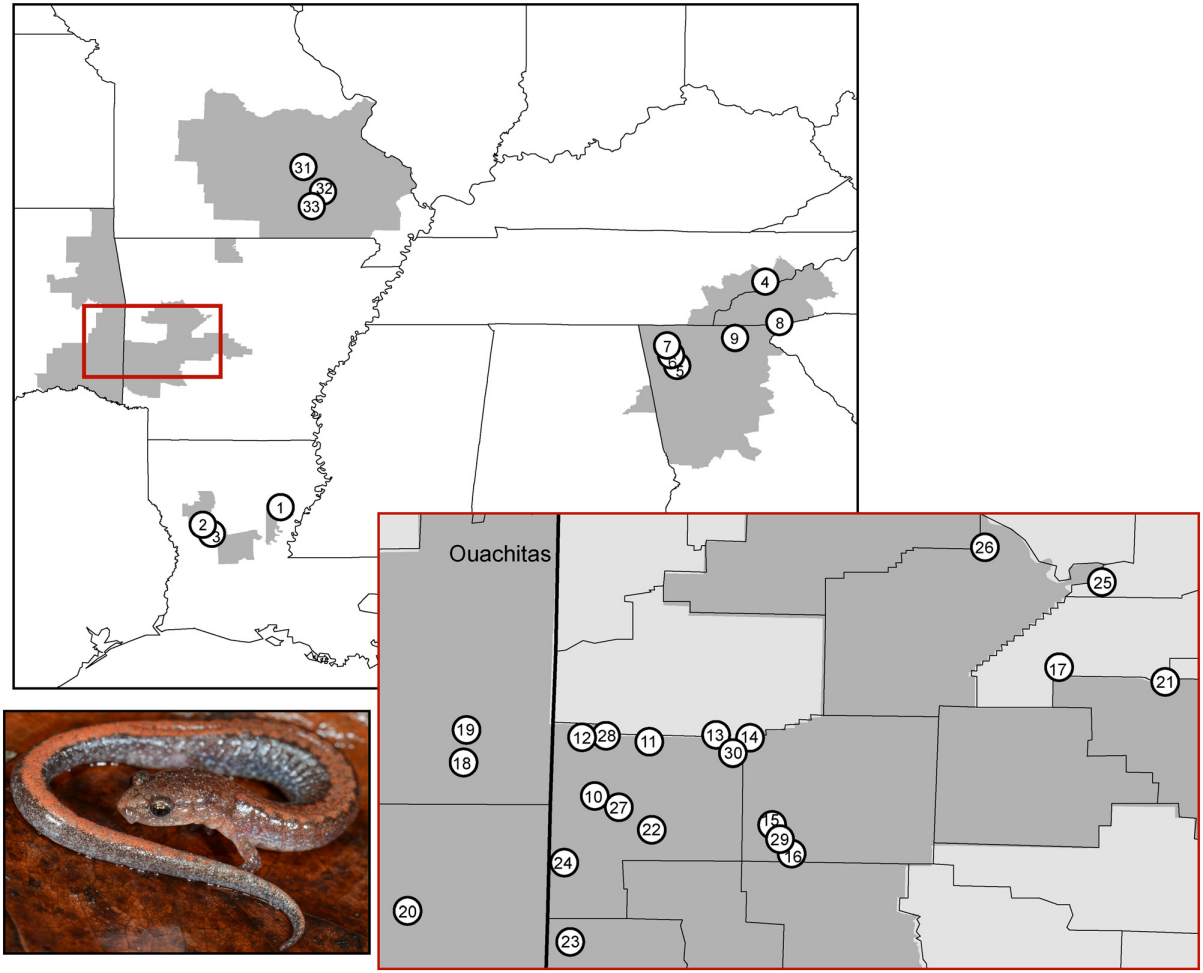
Map of collection localities. Numbers correspond to map code in Table 1. Inset: Ouachita region. OK = Oklahoma; AR = Arkansas. Photograph: *P*. *serratus*, LSUMZ 98343; photo credit: C.C.A.

Here, we use the terrestrial southern redback salamander, *Plethodon serratus*, as a case study to test the hypothesis that disjunct species ranges in the Southeast are climatic refugia for species that were more widely distributed during the LGM. *Plethodon serratus* is found in four isolated regions: the Ozark Mountains, the Ouachita Mountains, the Appalachian Mountains, and two parishes ( = counties) in Louisiana (Fig 1). The genus *Plethodon* of terrestrial woodland salamanders is the largest genus of salamanders in North America, with 55 species currently recognized and numerous cryptic species [15]. Within the eastern North American *Plethodon*, recent molecular studies place the *P*. *cinereus* group, of which *P*. *serratus* is a member, sister to all other eastern *Plethodon* [16–18]. Although intraspecific relationships of members of eastern *Plethodon* remain understudied (but see [19–23]), a recent survey of the mitochondrial relationships within *P*. *serratus* suggested that the systematics of this species may be more complex than indicated by current taxonomy, involving multiple genetic lineages without reciprocal monophyly of regions [24].

In this paper, we combine genetics and ecological niche modeling to test the following hypotheses: (1) the four disjunct regions of the *P*. *serratus* geographic range comprise independent evolutionary lineages, and (2) the geographic range of *P*. *serratus* was broader and more contiguous in the past and has since been restricted to the four regions by climate.

## Materials and Methods

### Sample collection

We included 208 tissues of *P*. *serratus* that we collected from the field or loaned from museums. Specimens were from 33 localities representing the entire species range (Table 1, Fig 1, S1 Table). We also included two specimens of the closely related species *P*. *cinereus* as an outgroup.

**Table 1:** Regions and populations sampled.

Population	Sample size	Map code	County	State
**Louisiana Region**				
Sicily Island WMA	15	1	Catahoula	Louisiana
Kisatchie Bayou	2	2	Natchitoches	Louisiana
Longleaf Vista	4	3	Natchitoches	Louisiana
**Appalachians Region**				
UTA Field Station	3	4	Sevier	Tennessee
Big Springs Branch	1	5	Gordon	Georgia
John's Creek	1	6	Floyd	Georgia
Furnace Creek	1	7	Walker	Georgia
Sunset Rocks Trail	1	8	Macon	North Carolina
Summertown	3	9	Gwinnett	Georgia
**Ouachitas Region**				
Iron Mountain	10	10	Polk	Arkansas
Foran Gap	9	11	Polk	Arkansas
Rich Mountain	20	12	Polk	Arkansas
Rich Mountain	2	12	Le Flore	Oklahoma
Fourche Mountain	14	13	Scott	Arkansas
Buck knob	13	14	Scott	Arkansas
Caddo Gap	12	15	Montgomery	Arkansas
County Rd 240	11	16	Montgomery	Arkansas
South Fourche	8	17	Perry	Arkansas
Kiamichi Mountain	5	18	Le Flore	Oklahoma
Winding Stair Mountain	3	19	Le Flore	Oklahoma
Beaver Bend State Park	21	20	McCurtain	Oklahoma
Ouachita Trail	1	21	Perry	Arkansas
Near Mena	6	22	Polk	Arkansas
DeQueen Lake	1	23	Sevier	Arkansas
Whiskey Peak	1	24	Polk	Arkansas
Petit Jean Mountain	1	25	Conway	Arkansas
Mount Nebo	3	26	Yell	Arkansas
Highway 74	1	27	Polk County	Arkansas
Black Fork Mountain	5	28	Polk	Arkansas
Polk Mountain	3	29	Montgomery	Arkansas
Brushy Knob	2	30	Polk	Arkansas
**Ozarks Region**				
Indian Trail	11	31	Dent	Missouri
Rocky Creek	10	32	Shannon	Missouri
Peck Ranch	10	33	Carter	Missouri

Map code corresponds to Fig 1.

### Ethics statement

All collecting by us was done under appropriate state collecting permits for Louisiana (Scientific Collecting Permits LNHP-13-036 and LNHP-14-010 and Wildlife Division Special Use Permit to Conduct Research on WMAs #WL-Research-2013-05). Collecting was conducted in strict accordance with a protocol approved by the Institutional Animal Care and Use Committee (IACUC) of Louisiana State University (permit number 13–060), which approved this complete study. The samples included in the present study are permanently held in the following repositories: Louisiana State University Museum of Natural Science, Museum of Vertebrate Zoology, Sam Noble Oklahoma Museum of Natural History, Sternberg Museum of Natural History, and the University of Alabama Herpetology Collection. All sample catalog numbers can be found in S1 Table.

### Genetic data collection

Genomic DNA was extracted using either Qiagen DNeasy Blood & Tissue Kits (Valencia, CA, USA) or a standard salt extraction protocol [25]. We amplified and sequenced a 728 bp segment of the mitochondrial cytochrome *b* (cytb) gene. We also amplified and sequenced six nuclear loci for a total of 3,256 bp: BDNF (667 bp), NCX1 (496 bp), POMC (465 bp), RAG-1 (663 bp), SLC8A3 (717 bp), and anonymous locus c3 (262 bp), which was developed for a phylogeographic study of another plethodontid genus, *Hydromantes* [26]. Primers, references, and annealing temperatures for each locus can be found in S2 Table. Sequences were visually verified and contigs assembled in Geneious v.6.0.5. All sequences were deposited in Genbank (accession numbers: KM883214-KM884672, S1 Table).

For each locus, sequences were aligned using the ClustalW algorithm in Geneious. We inferred individual alleles from degenerate sequences for each nuclear locus using Phase v.2.1.1 [27,28]. Sites that could not be inferred with a high posterior probability (>95%) were retained as missing data for downstream allelic analyses. The best-fit models of sequence evolution were estimated for each locus using jModelTest v.2.1.4 [29].

### Mitochondrial and concatenated phylogenetic analyses

For the mitochondrial cyt *b* locus, the phylogeny was estimated under Bayesian and maximum-likelihood (ML) frameworks. Bayesian analyses were performed in MrBayes v.3.2.2 [30] with the alignment partitioned by codon position. We conducted two runs of 10 million MCMC generations, with samples drawn every 5000 generations. Convergence was assessed in Tracer v.1.6 [31], ensuring that the likelihood score and other parameters had stabilized and that all effective sample sizes (ESSs) were >200. We discarded the first 25% of samples as burn-in. ML analyses were conducted in RAxML v.8.0.0 [32]. Nodal support was assessed with 1000 bootstrap pseudoreplicates. We calculated average pairwise Jukes-Cantor sequence divergence in DnaSP v.5.10.1 [33,34].

Individual gene trees also were estimated for each nuclear locus following the same procedures as above but without partitioning by codon position, using the unphased sequences. Descriptive statistics and tests for neutrality were calculated for each nuclear locus in Arlequin v. 3.5 [35].

### Cluster analyses and species tree reconstruction

While phylogenies reconstructed from concatenated data sets can be informative, they do not always reflect true evolutionary relationships, particularly in the presence of incomplete lineage sorting [36]. We therefore estimated the species tree for *P*. *serratus* under the multi-species coalescent in *BEAST v.2.1.3 [37]. Species tree analyses are often used to reconstruct phylogenetic relationships among a set of species, but these analyses can similarly be used with intraspecific data sets to reconstruct phylogenetic relationships among populations or other groups of individuals [36]. The latter scenario still requires *a priori* delimitation of “species,” which in this case we define as the populations that maximize Hardy-Weinberg equilibrium. We used a Bayesian clustering algorithm in Structure v.2.3.4 [38,39] to estimate the number of clusters (*K*) and the cluster assignments with the highest posterior probabilities. We implemented the admixture model [38], assumed correlation of allele frequencies [39], and utilized population of origin as prior information. For each *K* from 1 to 10, we ran 20 iterations, each consisting of 500,000 generations after a burn-in of 100,000 generations. The best estimate of *K* was determined by assessing the change in log-likelihood values between values of *K* [40] via the Structure Harvester web server [41]. The most likely set of cluster membership coefficients was determined in CLUMPP [42] using a greedy algorithm.

Structure grouped the Appalachians and Kisatchie samples in the same cluster with eastern Ouachita samples (see Results). However, the Appalachians, Kisatchie and eastern Ouachitas are separated by large geographic distances, and the nuclear phylogeny recovered an Appalachians clade. We therefore performed an additional species assignment test using Bayes factor delimitation (BFD) with path sampling in *BEAST. BFD uses estimated marginal likelihoods to compare multiple models of taxon assignment schemes. We tested two models: one model grouped Appalachians and Kisatchie with the eastern Ouachita samples as recovered by Structure, and the second model separated the Appalachian and Kisatchie samples into an additional two taxa. We excluded individuals that were not assigned to a Structure cluster with probability ≥ 0.9 [43]. We ran the path sampling analysis for 48 steps, with 50 million iterations for each step. The Bayes factor was calculated as twice the difference in marginal likelihood of the two models [44].

Using the preferred taxon scheme from the BFD analysis, we performed two species tree reconstructions: one with nuclear loci only and one with the nuclear and mitochondrial data. For each analysis, the starting tree was estimated under a Yule speciation model and uncorrelated lognormal relaxed clock for each locus. Each analysis was run for 250 million generations, sampling every 10,000 steps. Convergence was assessed in Tracer to ensure ESSs >200 after a burn-in of 20–50%.

### Ecological niche and paleodistribution modeling

To test for temporal changes in the geographic distribution of *P*. *serratus*, we used ecological niche modeling and paleodistribution modeling as implemented in Maxent v.3.3.3k [45]. Natural history collection specimen occurrence records for *P*. *serratus* were downloaded from online databases HerpNET (herpnet.org) and GBIF (gbif.org). A principle components analysis of the climate data extracted for each occurrence record showed that each region has a distinct climate (S1 Fig); we therefore built an ecological niche model (ENM) for each region independently. The ENMs were generated using 11 bioclimatic layers for temperature and precipitation (S3 Table) downloaded from Worldclim [46]. We selected this set of layers from the full set of 19 bioclimatic layers available from Worldclim based on the correlation analyses and biological rationales described in Rissler & Apodaca [47], developed for another plethodontid, *Aneides flavipunctatus*. These layers had a spatial resolution of 1 km^2^ and were based on weather station data from 1950–2000. Because presence-only modeling algorithms assume that pseudoabsences are drawn from areas with unsuitable climate [48], we clipped the climate layers to a rectangle limited to the extent of the region being modeled. For example, the layers used to build the ENM for the Appalachians was clipped to a rectangle that included only the Appalachians and excluded the other three regions of the species range. This method minimized the chance that pseudoabsences would be drawn from a region potentially suitable climatically yet inaccessible due to other, non-climatic factors. For each region, the ENM was then projected onto the full species range.

Paleodistribution models were generated for the last interglacial (LIG; ~120,000–140,000 YBP), the last glacial maximum (LGM; ~21,000 YBP), and the mid-Holocene (~6,000 YBP) by projecting the ENM for each region onto climate layers from those three time points. Bioclimatic layers for these periods were downloaded from Worldclim at a spatial resolution of 5 km^2^. LIG climate data were based on Otto-Bliesner et al. [49]. LGM and mid-Holocene climate data were based on two general circulation model simulations (available from http://pmip2.lsce.ipsl.fr): Community Climate System Model (CCSM) and Model for Interdisciplinary Research on Climate (MIROC). Additional information on the construction of these layers can be found on the Worldclim website (worldclim.org/downscaling). Paleodistribution models for the two LGM and two mid-Holocene data sets were averaged to generate a single model for each time point. We converted all ENMs and paleodistribution models to binary models using the threshold determined by Maxent that maximizes the sum of sensitivity and specificity. This method of threshold selection has been shown to be suitable when presence-only data are used [50].

We quantified pairwise overlap between ENMs in ENMTools [51] using the similarity test statistic *I*, based on the Hellinger distance. To determine whether observed niche differences were due to differences in habitat availability in each region (null hypothesis) or to differences in suitability or selection, we generated a null distribution of niche overlap using the background similarity analysis in ENMTools. For each pair of regions, a null distribution of *I* values was generated by comparing the ENM for region A to an ENM created from a set of random points from the background area for region B, defined as the area enclosed by a minimum convex polygon around the occurrences for region B, replicated 100 times. The number of random points was equivalent to the number of occurrences for region B in the original data set. Under a two-tailed test, a significant result would indicate niche conservatism or divergence.

To examine the importance of temperature versus precipitation in driving the differences between paleodistribution and current ENMs, and thus potential distribution shifts from the LGM to present, we used the presence (1) value of the binary paleodistribution model for the Appalachians as a constraint and generated 1000 random points within the bounds of the model. For each of those points, we extracted climate data from the LGM and current climate layers and ran a PCA to obtain a reduced number of uncorrelated variables and determine which variables, as indicated by their loadings, explain the majority of the variation in the data.

## Results

### Sequence data and phylogenetic analyses

**Fig 2:**
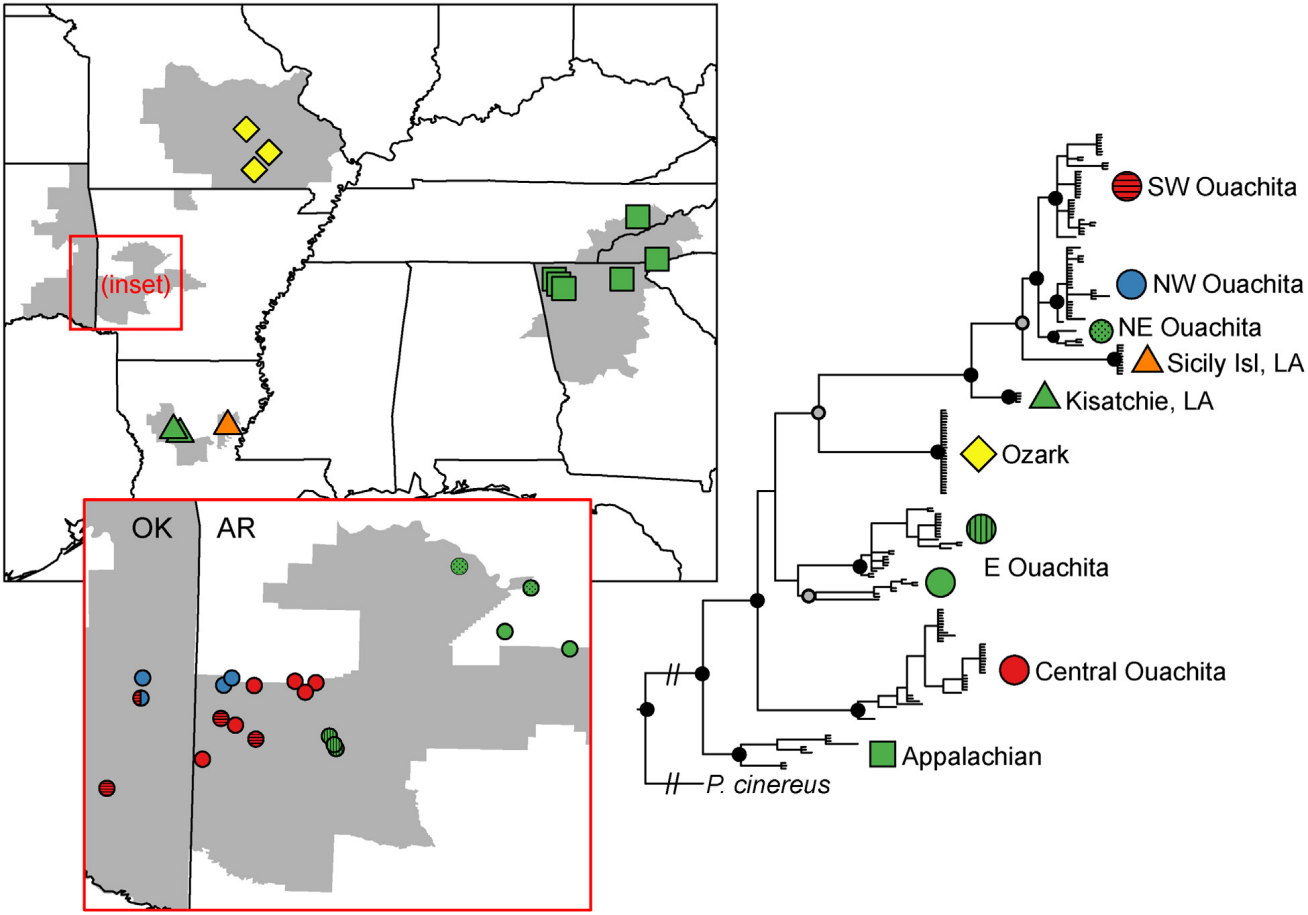
Maximum-likelihood phylogeny of mitochondrial cytb. Nodal support: grey dots: Bayesian PP > 0.9; black dots: ML bootstrap > 0.75 and Bayesian PP > 0.9. Shapes on the phylogeny correspond to map. Inset: Ouachita region.

The mitochondrial cyt *b* alignment was 728 bp long and contained 48 haplotypes. Average pairwise JC sequence divergence between geographic regions ranged from 4.4%-7.2%. Average JC sequence divergence within regions ranged from 0%-4.5% (Table 2). The ML phylogeny revealed 10 geographically concordant clades with strong support from ML bootstraps (≥ 75) and Bayesian posterior probabilities (≥ 0.9) (Fig 2, S2 Fig). The Appalachians and Ozarks each form strongly supported clades (Fig 2). Surprisingly, the two allopatric sites in Louisiana (Kisatchie, Sicily Island; Fig 1, Table 1) are not sister clades; rather, Sicily Island falls out sister to a clade comprised of samples from the Ouachitas. The Ouachita region as a whole also does not form a clade. To some extent, the spatial distribution of mitochondrial clades is concordant with geography at the population level, as populations that are closer together geographically tend to be more closely related. But this pattern does not hold at the larger scale, among regions, as only two of the four regions are represented by monophyletic clades.

**Table 2:** Average pairwise sequence divergence (JC) for mitochondrial cytb, among regions.

	Within Region (%)	Pairwise		
		Appalachians (%)	Ozarks (%)	Ouachitas (%)
Appalachians	1.3	-		
Ozarks	0.0	4.4	-	
Ouachitas	4.5	5.7	5.8	-
Louisiana	1.4	7.2	7.2	5.1

**Fig 3:**
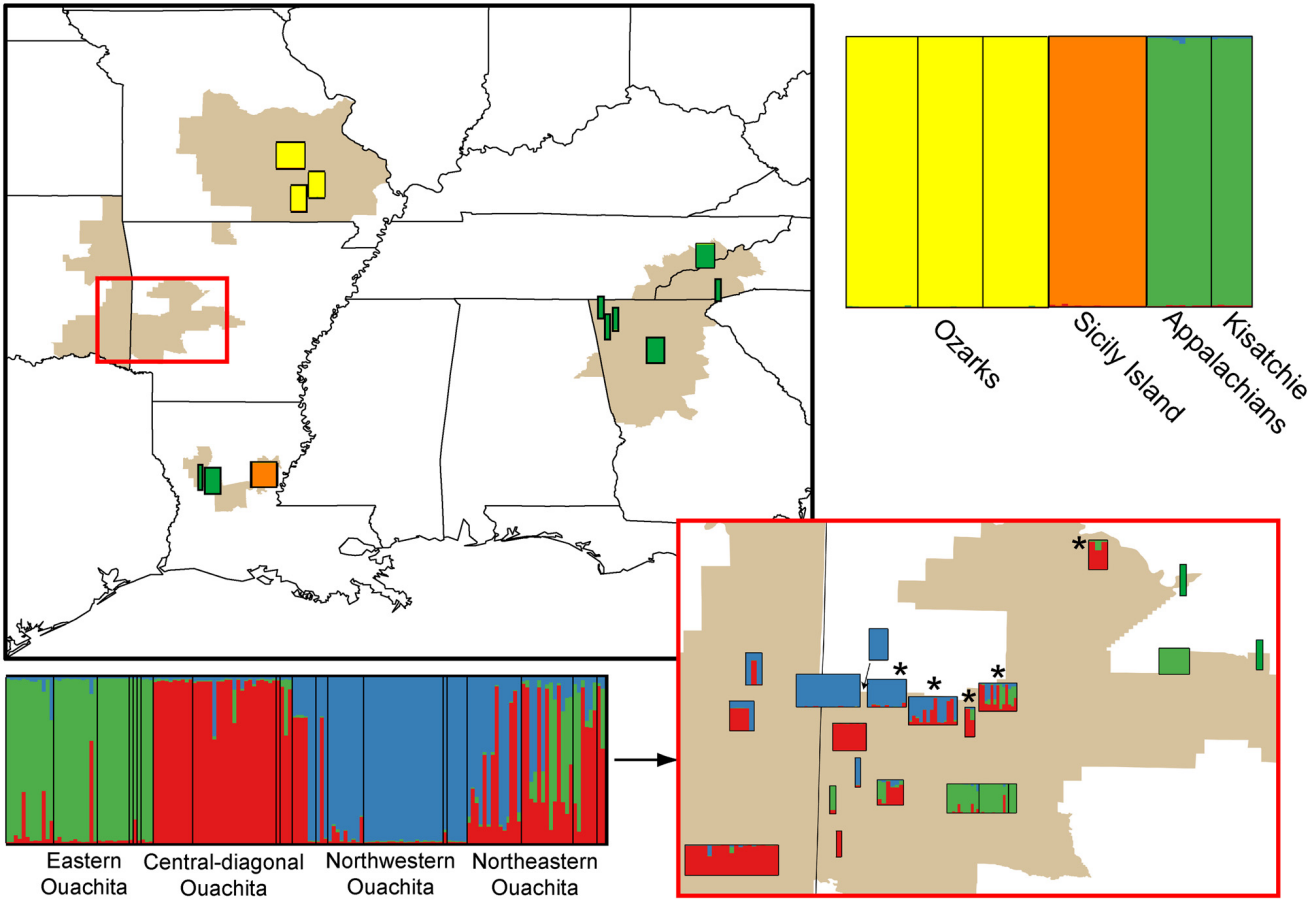
Structure clusters (K = 5) for the nuclear data set. Populations with individuals with the 9 bp deletion in the c3 locus are indicated by asterisks on the Ouachita inset. Colors and labels correspond to Fig 4.

The nuclear data sets consisted of a total of 3,270 bp. The number of variable sites for each locus ranged from 9–30 (S4 Table). Nucleotide diversity (π) and haplotype diversity (Hd) for each region are listed in S4 Table. Thirty of the 208 salamanders had a 9 bp deletion at anonymous nuclear locus c3; the deletion was present in 8 of 27 haplotypes for this locus. All 30 individuals possessing a haplotype with this deletion were from the northeastern Ouachitas (Fig 3, denoted by asterisks), from five populations: Foran Gap (2 samples of 9), Fourche Mountain (11 of 14), Brushy Knob (1 of 2), Buck Knob (13 of 13), Mt. Nebo (3 of 3). Eighteen of the 30 individuals were heterozygous for the deletion.

Individual nuclear gene trees (S3 Fig) showed little resolution, but, consistent with the mitochondrial phylogeny, the Ozarks, the Appalachians, and Sicily Island each form strongly supported clades for at least one locus. Structure recovered five clusters corresponding to (i) Ozarks, (ii) Sicily Island, (iii) Appalachian + Kisatchie + Ouachita, and (iv, v) two clusters unique to Ouachita (Fig 3). Admixture (*q* < 0.9) was prevalent among populations within the Ouachita region, whereas all individuals in the remaining three regions were assigned to a cluster with probability ≥ 0.9. Cluster assignment was not entirely concordant with the mitochondrial clades. Notably, Structure did not separate the Appalachian and Kisatchie samples from the eastern Ouachita, even with additional hierarchical runs (data not shown). Pairwise F_ST_ values for the Structure clusters ranged from 0.134 between two Ouachita clusters to 0.984 between the Ozark and Sicily Island clusters (Table 3). Pairwise F_ST_ values were highest for pairs that included the Ozarks and Sicily Island.

**Table 3:** Pairwise average F_ST_ values calculated from the concatenated nuclear data set.

	Ozark	Sicily Island	Kisatchie	Appalachian	E Ouach.	NW Ouach.
Ozark	-					
Sicily Island	0.9837	-				
Kisatchie	0.9720	0.9395	-			
Appalachian	0.9155	0.8602	0.5213	-		
E Ouach.	0.7902	0.7418	0.3778	0.4234	-	
NW Ouach.	0.7644	0.5948	0.5899	0.5762	0.4200	-
C Ouach.	0.7252	0.6093	0.4443	0.4789	0.2426	0.1337

All values are significant (p < 0.001).

**Fig 4:**
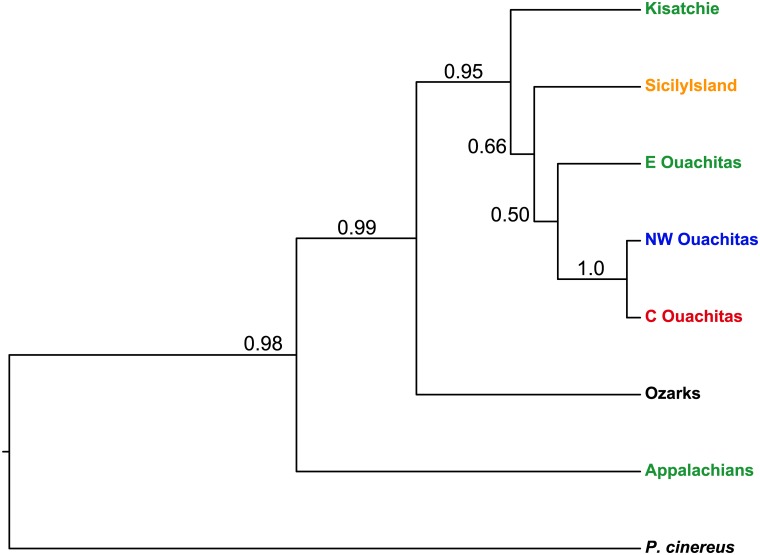
Species tree from *BEAST. Colors correspond to Structure bar plots in Fig 3. Nodal support: Bayesian PP.

Marginal likelihoods for the combined Appalachians/Kisatchie/E Ouachita model (-8567.4) and the separated model (-8447.4) yielded a Bayes factor of 239.9. Bayes factors greater than 10 indicate decisive support for one model over the other [44], so our BFD analysis strongly supports delimiting Appalachians and Kisatchie as separate taxa for the species tree analyses. For the species trees, the topologies and nodal support values for the nuclear-only and the combined nuclear and mitochondrial species trees were not qualitatively different, so we present only the species tree for the combined data set (Fig 4). Species tree analyses were largely congruent with the mitochondrial and nuclear phylogenies. The Appalachian region once again falls out sister to a strongly supported clade (PP = 0.99) consisting of all other samples. Unlike the mitochondrial tree, however, the species tree groups Ouachita and Louisiana into a single, strongly supported (PP = 0.95) clade, sister to the Ozark region.

### ENM and paleodistribution model analyses

**Fig 5:**
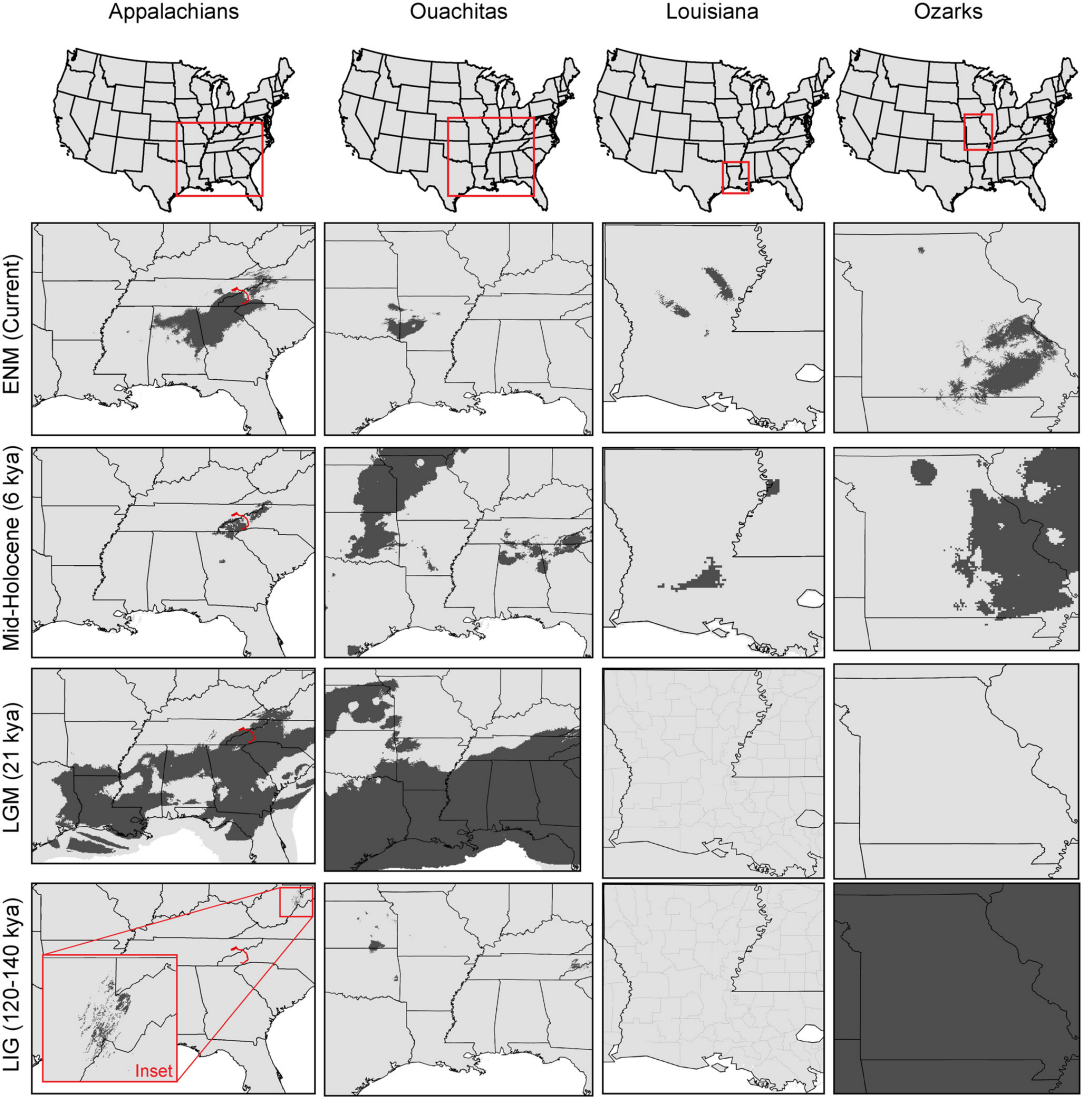
Ecological niche models (second row) and paleodistribution models (mid-Holocene, LGM, LIG). Columns: the four regions of the *P*. *serratus* range. Red line in the Appalachians denotes the French Broad River. For LGM models, note the expanded coastlines due to lower sea levels. For Ozark LIG model, note the area of suitability covers the entire depicted region.

The ENM generated for *P*. *serratus* roughly corresponded with the county based range map (IUCN et al., 2008), with overprediction beyond the current distribution in the Appalachian region (Fig 5). Niche similarity tests among the four regions showed all pairwise comparisons significantly more similar than expected based on chance. The LIG paleodistribution models for the Appalachians and Ouachitas both showed a very small area of suitable climate in the Appalachian highlands (Fig 5). The LIG model for Louisiana showed no areas of suitability, but the Ozark model found a vast area of suitability covering almost the entire eastern US and Canada (Fig 5). The LGM models for the Appalachians and Ouachitas showed a contiguous surface of suitable climate across the southeastern US, extending north to the Ouachitas but remaining south of the Ozarks (Fig 5). The LGM models for Louisiana and the Ozarks revealed no areas of suitable climate. Mid-Holocene models for the Appalachians and Ouachitas were much more restricted than the LGM models. Appalachian salamanders were restricted to the highlands, and Ouachita salamanders were restricted to an area encompassing parts of Oklahoma, Kansas, and Missouri. The mid-Holocene model for the Ozarks showed suitability in the Ozark Plateau and eastward into Illinois.

The PCA of paleo- and current climate at a set of random points within the hindcasted LGM range of the Appalachian region suggests that temperature is more important than precipitation in the distribution of *P*. *serratus*. PC1 explained 55.7% of the variation, and PC1 and PC2 combined explained 78.2% of the variation. PC1 is dominated by temperature variables (Bio1-11), and PC2 is dominated by precipitation variables (Bio12-18) (S3 Table).

## Discussion

Our ENM and paleodistribution model results support the hypothesis that *P*. *serratus* originated in the Appalachian Mountains and subsequently expanded southward and westward across the Coastal Plains during the cooler glacial periods. The high haplotype diversity (0.844) and nucleotide diversity (0.145) within the Appalachians further support the Appalachians as a source region. Recent phylogeographic studies of the spotted salamander *Ambystoma maculatum* found evidence of a similar pattern of expansion from the Appalachians along the Coastal Plains, and north to the Interior Highlands of the Ouachitas and Ozarks [52–54]. In addition, Phillips [52] found very low genetic diversity within the Ozarks, mirroring our results for *P*. *serratus*. In the Ozarks, we recovered only one mitochondrial haplotype, and again only one haplotype for four of the six nuclear loci. This low genetic diversity, combined with the lack of suitable habitat in the Ozarks during the LGM, suggests that this region was more recently colonized.

The LIG paleodistribution model for the Ozarks suggests a vast area of suitable habitat across the entire eastern North America (Fig 5). However, because none of our other results indicate a widespread Ozark lineage, we cautiously suggest two alternative explanations for this result. First, it is possible that this result is an artifact of the spatial resolution of the climate layers, the small sample size, or both. However, Maxent has been shown to be robust to small sample sizes [55], and the AUC scores for this model were consistently high across multiple replicate runs. Therefore, we suggest the possibility that although the current habitat for the Ozark lineage may have been widespread during the LIG, the region had not yet been colonized. Paleodistribution models for the subsequent time period, the LGM, provide additional support for this scenario.

*Plethodon serratus* salamanders in the four regions are separated by large geographic distances, and *P*. *serratus* has relatively poor dispersal ability. We would thus predict deep genetic divergence in the mitochondrial loci with clear geographic concordance if the four regions have been isolated long-term. Instead, however, mitochondrial and nuclear haplotypes are shared among regions. Mitochondrial results show geographic structure but, particularly in the Ouachitas, lack the signature of reciprocal monophyly with deep genetic divergence suggestive of persistent isolation. In the mitochondrial phylogeny, the Louisiana samples are nested within the Ouachitas, and the Kisatchie and Sicily Island clades are not sister to each other (Fig 2). Furthermore, while the geographic distributions of the mitochondrial haplotypes and Structure clusters from the nuclear data are largely congruent, there is some discordance. For example, the three samples from Mt. Nebo, AR, fell out in a clade with the other eastern Ouachita samples in the mitochondrial phylogeny (Fig 2) but clustered with the central and southwestern Ouachita samples in the Structure analysis (Fig 3).

Possible explanations for these phylogenetic patterns include incomplete lineage sorting and introgression of mitochondrial haplotypes. Given the ENMs and paleodistribution models, we suggest the former scenario is the more plausible one. We would expect some level of incomplete lineage sorting in a scenario of past range expansion followed by recent contraction and isolation. Populations generally gradually progress from polyphyly and paraphyly to reciprocal monophyly following geographic isolation [56]. Although recent migration and incomplete lineage sorting can result in similar genetic signals, ongoing migration among the four regions of the *P*. *serratus* range is unlikely for a salamander with such limited vagility. Moreover, the geographic distances separating the regions are large, and the intervening areas contain inhospitable habitat. However, we cannot rule out ongoing migration within regions, such as among populations within the Ouachitas. The ENM for the Ouachitas does not indicate unsuitable habitat in the valleys separating various mountain ranges inhabited by *P*. *serratus*, but we cautiously note that the 1 km^2^ resolution of the climate layers used to generate the ENMs may be too coarse to assess climate suitability at such a fine scale.

*Plethodon serratus* is fully terrestrial, lacking an aquatic larval stage, and thus is not dependent on creeks or vernal pools for breeding. We would expect, therefore, for the geographic distribution of *P*. *serratus* to be driven more by changes in temperature than precipitation. This is evident in the PCA scatterplot of paleo- and current climate (S4 Fig), where the primary PC axis, explaining 55.7% of the variation, is most heavily weighted by temperature. The two time points appear to overlap substantially along the y-axis, which is dominated by precipitation variables. These data suggest that *P*. *serratus* has expanded and contracted its range with temperature changes, retreating to the Interior Highlands and Appalachians during warmer periods. During the Pleistocene, the Coastal Plain was dominated by pine and oak, with cooler temperatures than today and much less precipitation [57]. The Southeast during the mid-Holocene was much warmer than the LGM [58], which may have driven the range contraction observed in the Appalachians and Ouachitas (Fig 5). This hypothesis of range expansion and contraction can be explicitly tested with a larger genetic data set by estimating changes in effective population size through time, employing coalescent analyses such as the Bayesian skyline plot [59].

This scenario does not explain why *P*. *serratus* is found in Louisiana, a region with much warmer current temperatures than all other localities in its range (S1 Fig). The fact that salamanders from the two general sites in Louisiana (Kisatchie and Sicily Island) are not each other’s closest relative suggests at least two independent colonizations of Louisiana by this species. Our analyses of niche similarity show that the ecological niches of the salamanders in each of the four regions are significantly non-identical, but there is no evidence that these differences are due to habitat selection or suitability differences rather than to an artifact of differences in the habitat available in each region [51]. Instead, results show ENMs to be more similar than expected by random sampling of the environment, which may suggest niche conservatism, but may also be an artifact of Brownian motion-like evolution of the niches [60]. Alternatively, this result may simply be a consequence of allopatric diversification and subsequent range shifts [61]. Therefore, additional studies on the ecology and behavior of *P*. *serratus* are necessary to determine the extent to which *P*. *serratus* is able to adapt in situ to changes in climate.

The Appalachian ENM for *P*. *serratus* overpredicts west into Alabama, north farther into North Carolina, and east into South Carolina (Fig 5), suggesting that factors other than climate are also driving the species distribution in the Appalachians. *Plethodon serratus* is replaced by *P*. *cinereus* to the northeast. The two species occur within 50 km of each other at their closest known sites, on opposite sides of the French Broad River Valley [62], with *P*. *dorsalis* occurring in the intervening regions. On the ENM, the narrow gap between the predicted distribution of *P*. *serratus* and the overpredicted area outside the species range to the northeast corresponds to the French Broad River Valley (Fig 5). This river is a known phylogenetic break in other species of plethodontid salamanders (*Desmognathus wrighti*; [63]) due to the inhospitable habitat in this intervening region. It is thus likely that *P*. *serratus* is restricted to the northeast by the French Broad River rather than by interactions with *P*. *cinereus*.

It is unclear why the ENM overpredicts to the west into Alabama. One possibility is that one or more specimens collected from eastern Alabama and included in our ENM analyses were misidentified as *P*. *serratus*. Alternatively, *P*. *serratus* may be restricted in this region by interspecific interactions. The congeners *P*. *websteri* and *P*. *dorsalis* also occur in Alabama. Competitive interactions of *P*. *serratus* have not been studied, but *P*. *serratus* and *P*. *ventralis* (sister to *P*. *dorsalis*) are known to replace each other altitudinally in the Appalachians, with *P*. *serratus* restricted to higher elevations where the two species co-occur [64]. It has been previously shown that species range shapes tend to be determined by a combination of climate, dispersal limitations, and interspecific competition [65], but we note that the overprediction may also simply be an artifact of the modeling algorithm or the suite of climate variables used to construct the ENM.

Our understanding of the ecology and evolutionary history of *P*. *serratus* is especially vital in Louisiana, where the species is listed as Critically Imperiled by the Louisiana Department of Wildlife and Fisheries. *Plethodon serratus* is currently restricted to two known localities in Louisiana: the Longleaf Vista Outlook in the Kisatchie National Forest, and Sicily Island Hills WMA [66]. In addition, habitat destruction from strip mining in the state has resulted in the likely extirpation of at least one isolated population in DeSoto Parish [67]. However, because the Longleaf Vista site is located along a heavily used public trail, it seems likely that this restricted distribution is at least partially a result of sampling bias and that *P*. *serratus* may also occur elsewhere in the Kisatchie National Forest. Other localities in this area also contain habitat more similar to the mixed hardwood forest of Sicily Island than to the pine and sandstone habitat at Longleaf Vista. At one locality west of Longleaf Vista, we captured two *P*. *serratus* (Tables 1 and S1). This is a previously undocumented locality for this species. The samples from the new locality shared mitochondrial and nuclear haplotypes with those from Longleaf Vista. It is unclear if one of those two sites is a recent colonization by *P*. *serratus* or if the species is able to readily migrate between the two sites. Although our discovery encourages hope that *P*. *serratus* may be more abundant in Louisiana than previously thought, we caution that the species is still known only from a small area within the Kisatchie National Forest and from Sicily Island. We further highlight the need for additional surveys of *P*. *serratus* in the area.

### Conclusions

Our study underscores the power of synthesizing information from genetics and climate to uncover factors driving species distributions. Our results suggest that *P*. *serratus* was much more broadly distributed across the Coastal Plain during the LGM and has contracted to its current disjunct range in response to warming. However, our study also highlights the importance of understanding the variation in individual responses to climate among even closely related species. *Plethodon serratus* appears to be unique within the genus in flourishing broadly during historical cooler climates, as other members of *Plethodon* show a pattern of contraction (e.g., *P*. *caddoensis*; [22]). For *P*. *serratus*, an understanding of how the species responds to changes in climate may be vital to its survival in the future. We suggest that *P*. *serratus* alters its range more in response to temperature than to precipitation and that warmer temperatures lead to range contractions and further isolation. As global temperatures are predicted to continue to rise over the next 100 years [68], management of this species may be necessary in order to prevent further loss of genetic diversity or extinction, especially in Louisiana, where its current distribution is the most restricted and fragmented. Additional research is also needed exploring the comparative phylogeography of the Southeastern U.S. to determine the extent to which the biogeographic and evolutionary processes revealed in this study can be generalized to other amphibian species in the region. Larger genetic data sets and a taxonomically more inclusive paleodistribution model study will allow for explicit testing under a coalescent framework of the hypotheses presented in this study and, more generally, provide further insight into amphibian responses to historical climatic changes and potential consequences of future warming.

## Supporting Information

S1 Fig Scatterplot of climate data extracted from current Worldclim layers. Climate data were extracted for all *P*. *serratus* localities included in the ENM analyses.
(PDF)

S2 Fig ML phylogeny of mitochondrial cytb, with individual labels. Tree is identical to Fig 2, except tip labels are retained. Tip labels correspond to S1 Table.
(PDF)

S3 Fig Individual gene trees for the nuclear loci. Trees were generated under a maximum-likelihood framework. Nodal support: Bayesian PP/ML bootstraps. Tip labels correspond to S1 Table.
(PDF)

S4 Fig PCA of current vs. LGM climate data. Climate data were extracted from 1,000 random points in the retrodicted LGM distribution of Appalachian *P*. *serratus*. Black: LGM climate data, red: current climate data for the same localities.
(PDF)

S1 Table List of specimens included in study. Associated collection information and Genbank Accession numbers.
(XLSX)

S2 Table Primer information. Primer names, reference citations, sequences, and annealing temperatures used in genetic analyses.
(XLSX)

S3 Table Eigenvalues corresponding to S4 Fig. (XLS)

S4 Table Descriptive statistics and tests of neutrality for nuclear loci. (XLSX)
