# Supplementary figures and images for: Thriving in the Cold: Glacial Expansion and Post-Glacial Contraction of a Temperate Terrestrial Salamander (Plethodon serratus)
Source: PLoS One. 2015 Jul 1;10(7):e0130131. doi: 10.1371/journal.pone.0130131 (PMC4488858; doi:10.1371/journal.pone.0130131)

Principle Component Analysis of Climate Variables

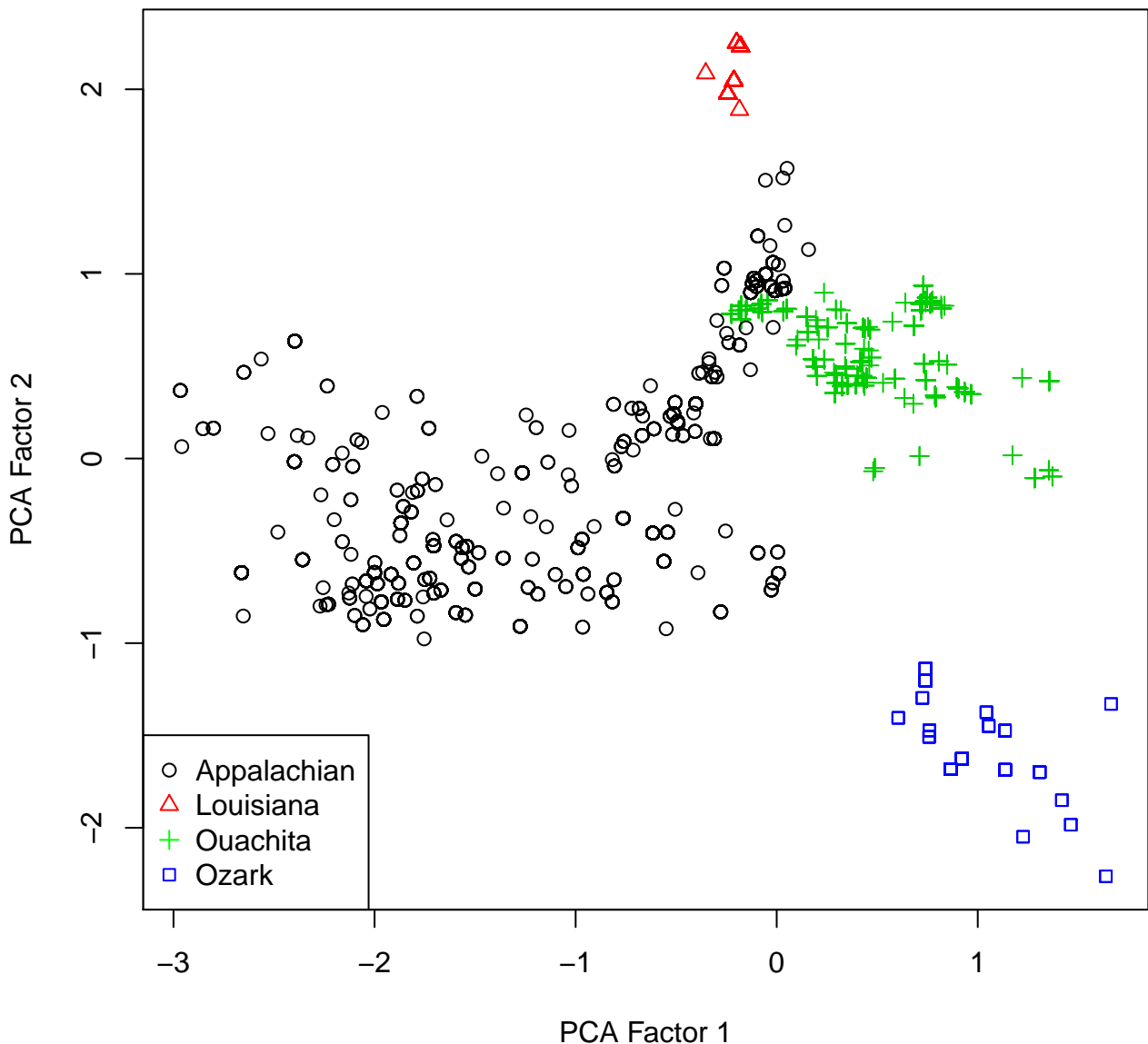

Supplement: S1 Fig — Climate data were extracted for all P. serratus localities included in the ENM analyses. (PDF) [file pone.0130131.s001.pdf]

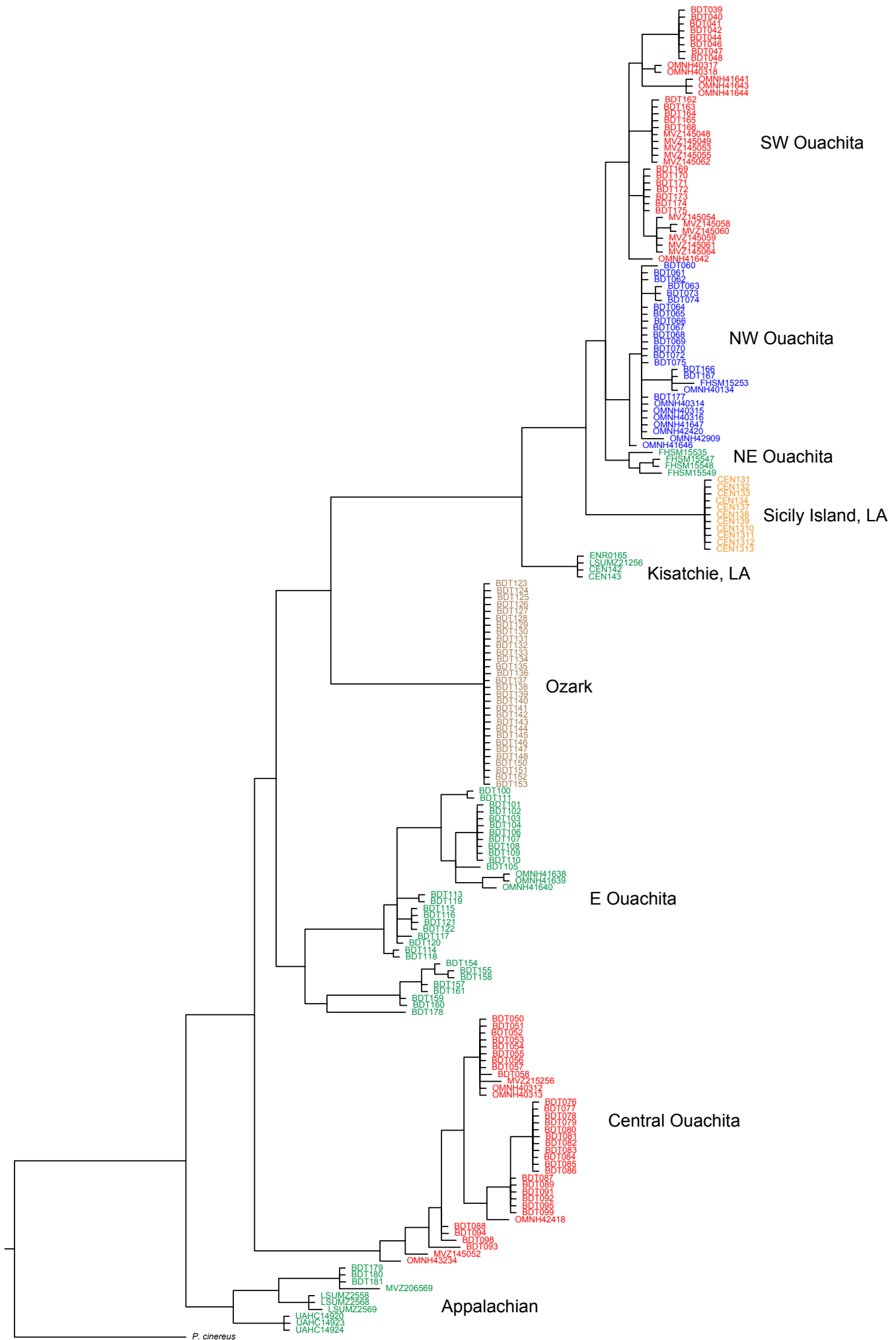

Supplement: S2 Fig — Tree is identical to Fig 2, except tip labels are retained. Tip labels correspond to S1 Table. (PDF) [file pone.0130131.s002.pdf]

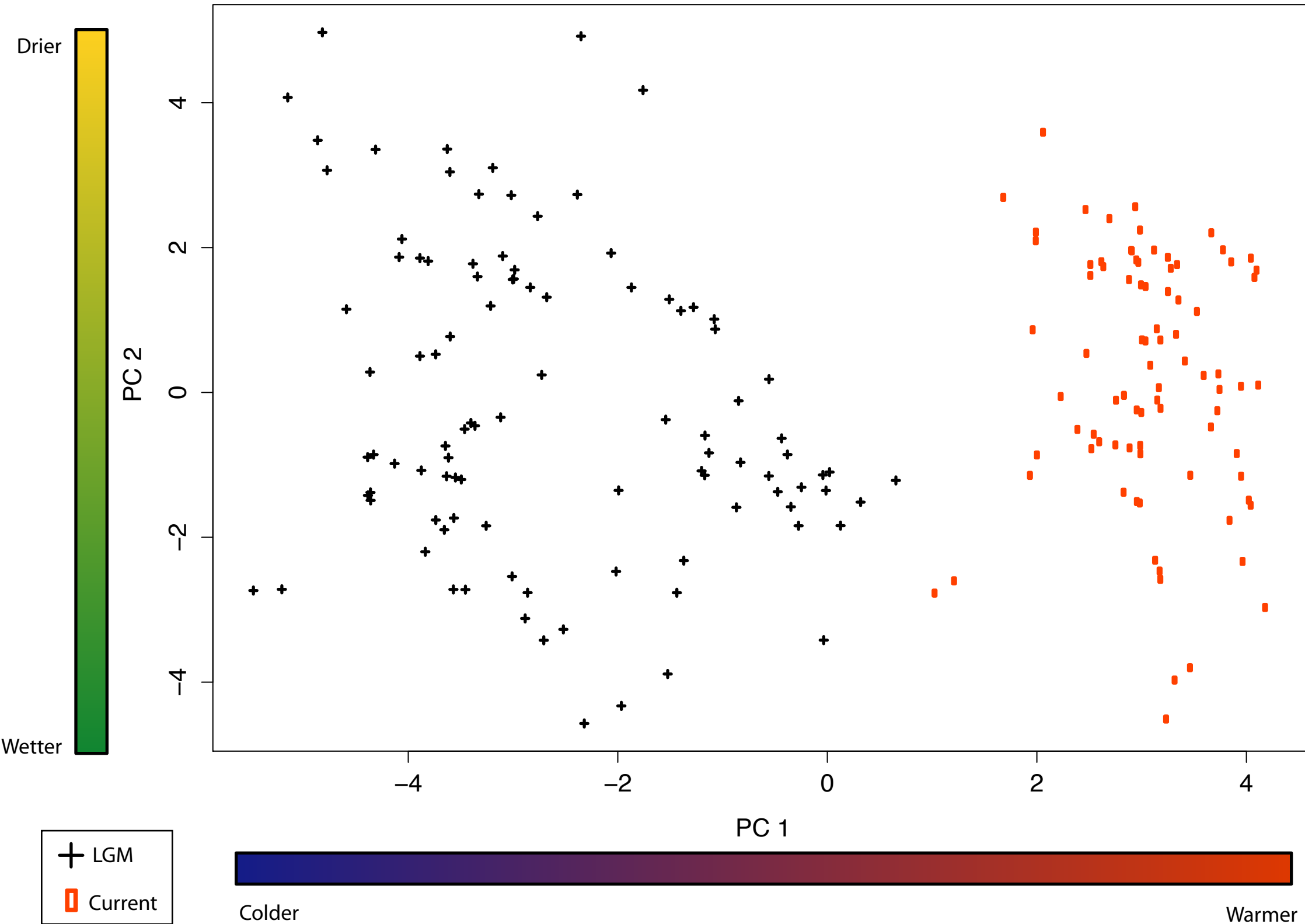

Supplement: S4 Fig — Climate data were extracted from 1,000 random points in the retrodicted LGM distribution of Appalachian P. serratus. Black: LGM climate data, red: current climate data for the same localities. (PDF) [file pone.0130131.s004.pdf]
