# Supplementary material for: Thriving in the Cold: Glacial Expansion and Post-Glacial Contraction of a Temperate Terrestrial Salamander (Plethodon serratus)
Source: PLoS One. 2015 Jul 1;10(7):e0130131. doi: 10.1371/journal.pone.0130131 (PMC4488858; doi:10.1371/journal.pone.0130131)
Supplement: S3 Fig — Trees were generated under a maximum-likelihood framework. Nodal support: Bayesian PP/ML bootstraps. Tip labels correspond to S1 Table. (PDF) [file pone.0130131.s003.pdf]

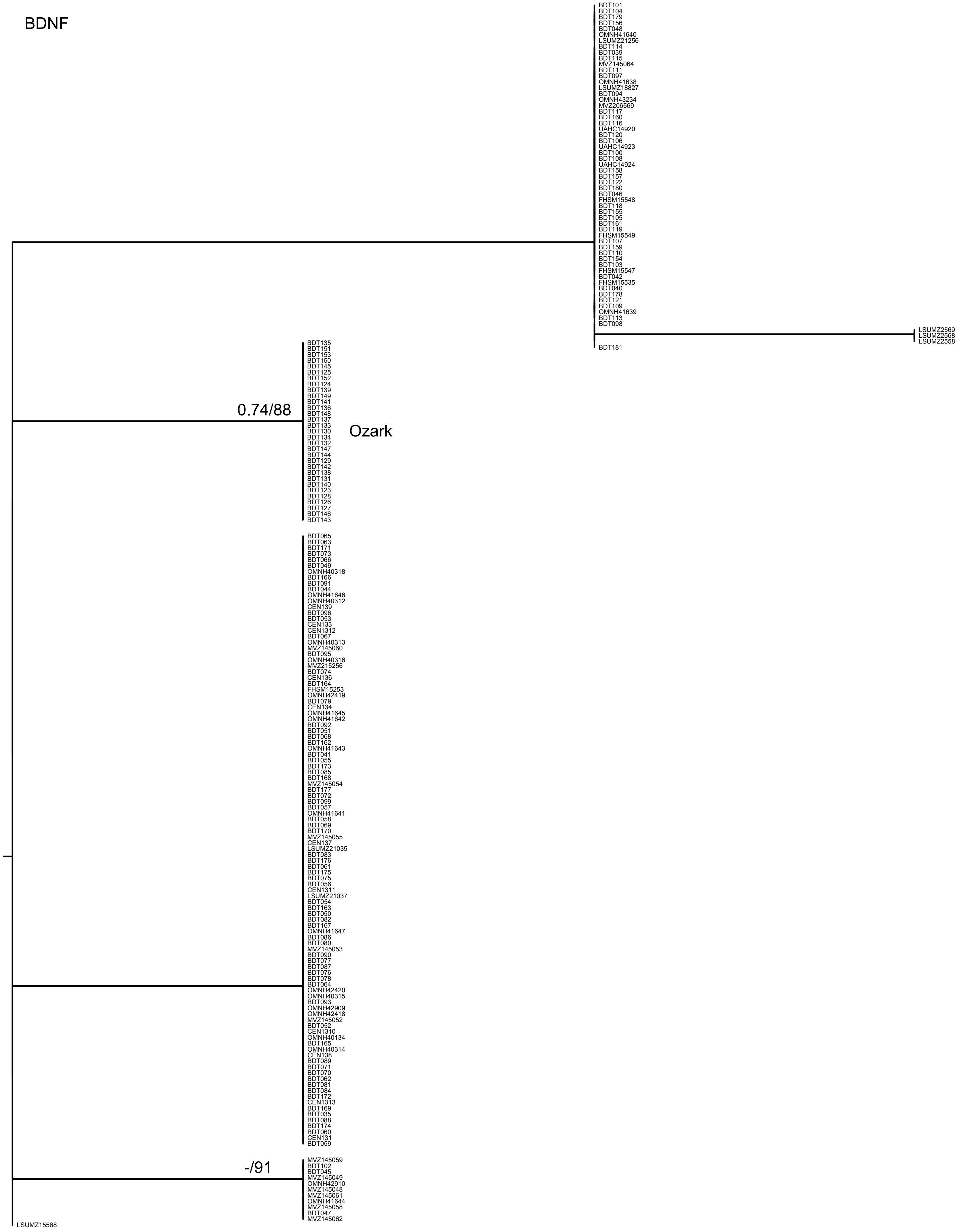
BDNF
BDT101
BDT104
BDT179
BDT156
BDT048
OMNH41640
LSUMZ21256
BDT114
BDT039
BDT115
MVZ145064
BDT111
BDT097
OMNH41638
LSUMZ18827
BDT094
OMNH43234
MVZ206569
BDT117
BDT160
BDT116
UAHC14920
BDT120
BDT106
UAHC14923
BDT100
BDT108
UAHC14924
BDT158
BDT157
BDT122
BDT180
BDT046
FHSM15548
BDT118
BDT155
BDT105
BDT161
BDT119
FHSM15549
BDT107
BDT159
BDT110
BDT154
BDT103
FHSM15547
BDT042
FHSM15535
BDT040
BDT178
BDT121
BDT109
OMNH41639
BDT113
BDT098
LSUMZ2569
LSUMZ2568
LSUMZ2558
BDT181
BDT135
BDT151
BDT153
BDT150
BDT145
BDT125
BDT152
BDT124
BDT139
BDT149
BDT141
BDT136
BDT148
BDT137
BDT133
BDT130
BDT134
BDT132
BDT147
BDT144
BDT129
BDT142
BDT138
BDT131
BDT140
BDT123
BDT128
BDT126
BDT127
BDT146
BDT143
0.74/88
Ozark
BDT065
BDT063
BDT171
BDT073
BDT066
BDT049
OMNH40318
BDT166
BDT091
BDT044
OMNH41646
OMNH40312
CEN139
BDT096
BDT053
CEN133
CEN1312
BDT067
OMNH40313
MVZ145060
BDT095
OMNH40316
MVZ215256
BDT074
CEN136
BDT164
FHSM15253
OMNH42419
BDT079
CEN134
OMNH41645
OMNH41642
BDT092
BDT051
BDT068
BDT162
OMNH41643
BDT041
BDT055
BDT173
BDT085
BDT168
MVZ145054
BDT177
BDT072
BDT099
BDT057
OMNH41641
BDT058
BDT069
BDT170
MVZ145055
CEN137
LSUMZ21035
BDT083
BDT176
BDT061
BDT175
BDT075
BDT056
CEN1311
LSUMZ21037
BDT054
BDT163
BDT050
BDT082
BDT167
OMNH41647
BDT086
BDT080
MVZ145053
BDT090
BDT077
BDT087
BDT076
BDT078
BDT064
OMNH42420
OMNH40315
BDT093
OMNH42909
OMNH42418
MVZ145052
BDT052
CEN1310
OMNH40134
BDT165
OMNH40314
CEN138
BDT089
BDT071
BDT070
BDT062
BDT081
BDT084
BDT172
CEN1313
BDT169
BDT035
BDT088
BDT174
BDT060
CEN131
BDT059
-/91
MVZ145059
BDT102
BDT045
MVZ145049
OMNH42910
MVZ145048
MVZ145061
OMNH41644
MVZ145058
BDT047
MVZ145062
LSUMZ15568

c3

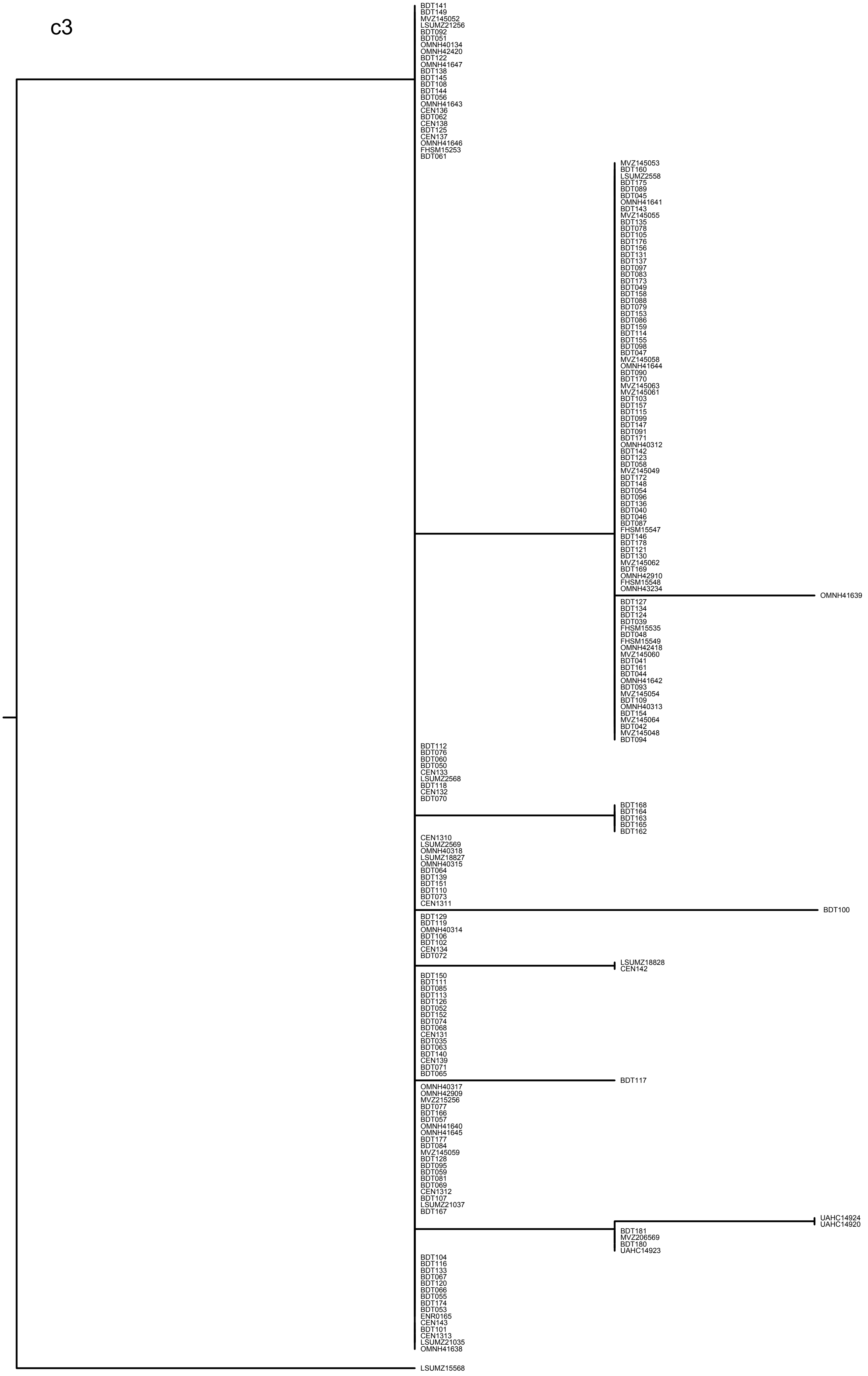
BDT141
BDT149
MVZ145052
LSUMZ21256
BDT092
BDT051
OMNH40134
OMNH42420
BDT122
OMNH41647
BDT138
BDT145
BDT108
BDT144
BDT056
OMNH41643
CEN136
BDT062
CEN138
BDT125
CEN137
OMNH41646
FHSM15253
BDT061
MVZ145053
BDT160
LSUMZ2558
BDT175
BDT089
BDT045
OMNH41641
BDT143
MVZ145055
BDT135
BDT078
BDT105
BDT176
BDT156
BDT131
BDT137
BDT097
BDT083
BDT173
BDT049
BDT158
BDT088
BDT079
BDT153
BDT086
BDT159
BDT114
BDT155
BDT098
BDT047
MVZ145058
OMNH41644
BDT090
BDT170
MVZ145063
MVZ145061
BDT103
BDT157
BDT115
BDT099
BDT147
BDT091
BDT171
OMNH40312
BDT142
BDT123
BDT058
MVZ145049
BDT172
BDT148
BDT054
BDT096
BDT136
BDT040
BDT046
BDT087
FHSM15547
BDT146
BDT178
BDT121
BDT130
MVZ145062
BDT169
OMNH42910
FHSM15548
OMNH43234
OMNH41639
BDT127
BDT134
BDT124
BDT039
FHSM15535
BDT048
FHSM15549
OMNH42418
MVZ145060
BDT041
BDT161
BDT044
OMNH41642
BDT093
MVZ145054
BDT109
OMNH40313
BDT154
MVZ145064
BDT042
MVZ145048
BDT094
BDT112
BDT076
BDT060
BDT050
CEN133
LSUMZ2568
BDT118
CEN132
BDT070
BDT168
BDT164
BDT163
BDT165
BDT162
CEN1310
LSUMZ2569
OMNH40318
LSUMZ18827
OMNH40315
BDT064
BDT139
BDT151
BDT110
BDT073
CEN1311
BDT100
BDT129
BDT119
OMNH40314
BDT106
BDT102
CEN134
BDT072
LSUMZ18828
CEN142
BDT150
BDT111
BDT085
BDT113
BDT126
BDT052
BDT152
BDT074
BDT068
CEN131
BDT035
BDT063
BDT140
CEN139
BDT071
BDT065
BDT117
OMNH40317
OMNH42909
MVZ215256
BDT077
BDT166
BDT057
OMNH41640
OMNH41645
BDT177
BDT084
MVZ145059
BDT128
BDT095
BDT059
BDT081
BDT069
CEN1312
BDT107
LSUMZ21037
BDT167
UAHC14924
UAHC14920
BDT181
MVZ206569
BDT180
UAHC14923
BDT104
BDT116
BDT133
BDT067
BDT120
BDT066
BDT055
BDT174
BDT053
ENR0165
CEN143
BDT101
CEN1313
LSUMZ21035
OMNH41638
LSUMZ15568

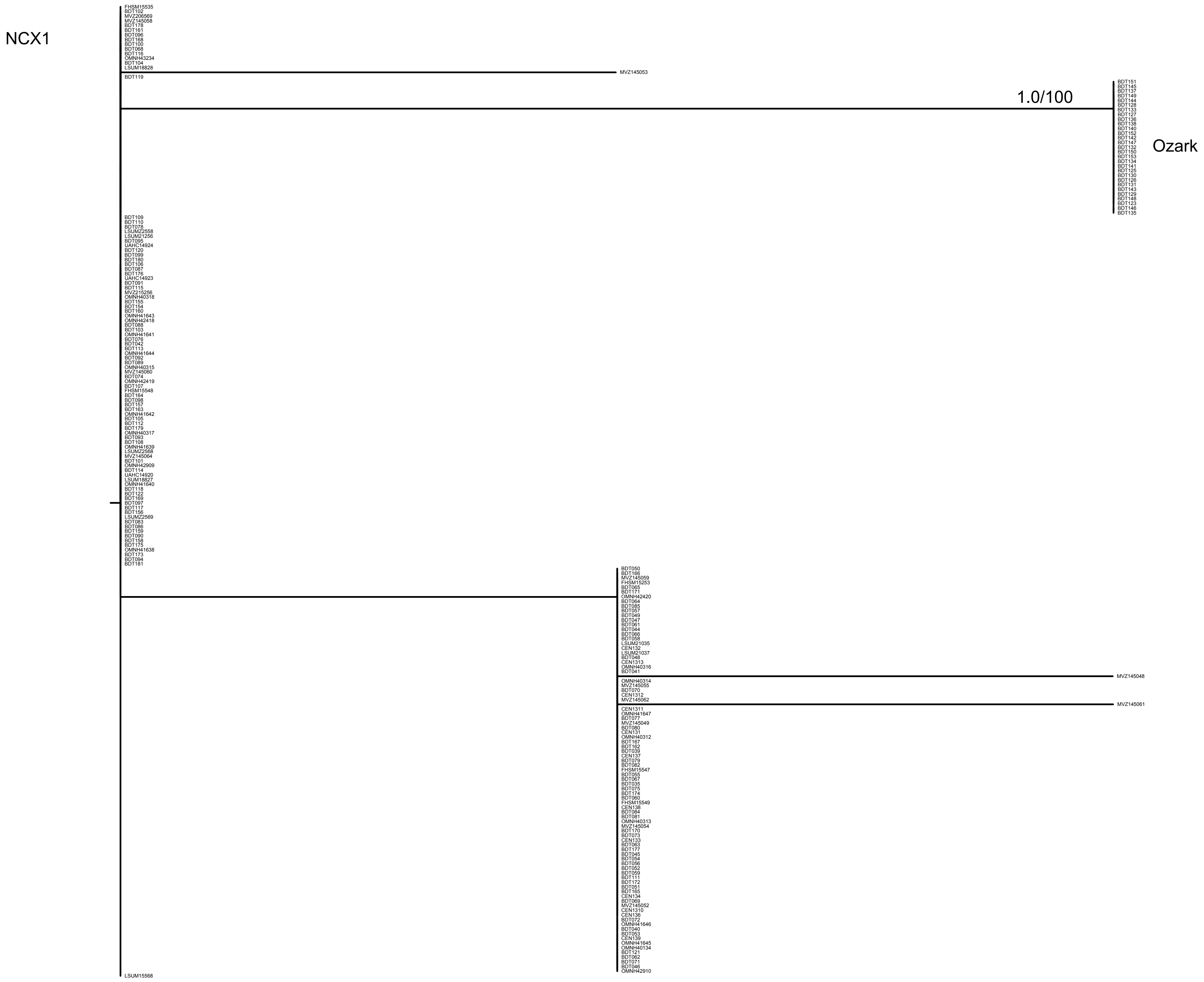

NCX1
FHSM15535
BDT102
MVZ206569
MVZ145058
BDT178
BDT161
BDT096
BDT168
BDT100
BDT068
BDT116
OMNH43234
BDT104
LSUM18828
MVZ145053
BDT119
1.0/100
BDT151
BDT145
BDT137
BDT149
BDT144
BDT128
BDT133
BDT127
BDT136
BDT138
BDT140
BDT152
BDT142
BDT147
BDT132
BDT150
BDT153
BDT134
BDT141
BDT125
BDT130
BDT126
BDT131
BDT143
BDT129
BDT148
BDT123
BDT146
BDT135
Ozark
BDT109
BDT110
BDT078
LSUMZ2558
LSUM21256
BDT095
UAHC14924
BDT120
BDT099
BDT180
BDT106
BDT087
BDT176
UAHC14923
BDT091
BDT115
MVZ215256
OMNH40318
BDT155
BDT154
BDT160
OMNH41643
OMNH42418
BDT088
BDT103
OMNH41641
BDT076
BDT042
BDT113
OMNH41644
BDT092
BDT089
OMNH40315
MVZ145060
BDT074
OMNH42419
BDT107
FHSM15548
BDT164
BDT098
BDT157
BDT163
OMNH41642
BDT105
BDT112
BDT179
OMNH40317
BDT093
BDT108
OMNH41639
LSUMZ2568
MVZ145064
BDT101
OMNH42909
BDT114
UAHC14920
LSUM18827
OMNH41640
BDT118
BDT122
BDT169
BDT097
BDT117
BDT156
LSUMZ2569
BDT083
BDT086
BDT159
BDT090
BDT158
BDT175
OMNH41638
BDT173
BDT094
BDT181
BDT050
BDT166
MVZ145059
FHSM15253
BDT065
BDT171
OMNH42420
BDT064
BDT085
BDT057
BDT049
BDT047
BDT061
BDT044
BDT066
BDT058
LSUM21035
CEN132
LSUM21037
BDT048
CEN1313
OMNH40316
BDT041
MVZ145048
OMNH40314
MVZ145055
BDT070
CEN1312
MVZ145062
MVZ145061
CEN1311
OMNH41647
BDT077
MVZ145049
BDT080
CEN131
OMNH40312
BDT167
BDT162
BDT039
CEN137
BDT079
BDT082
FHSM15547
BDT055
BDT067
BDT035
BDT075
BDT174
BDT060
FHSM15549
CEN138
BDT084
BDT081
OMNH40313
MVZ145054
BDT170
BDT073
CEN133
BDT063
BDT177
BDT045
BDT054
BDT056
BDT052
BDT059
BDT111
BDT172
BDT051
BDT165
CEN134
BDT069
MVZ145052
CEN1310
CEN136
BDT072
OMNH41646
BDT040
BDT053
CEN139
OMNH41645
OMNH40134
BDT121
BDT062
BDT071
BDT046
OMNH42910
LSUM15568

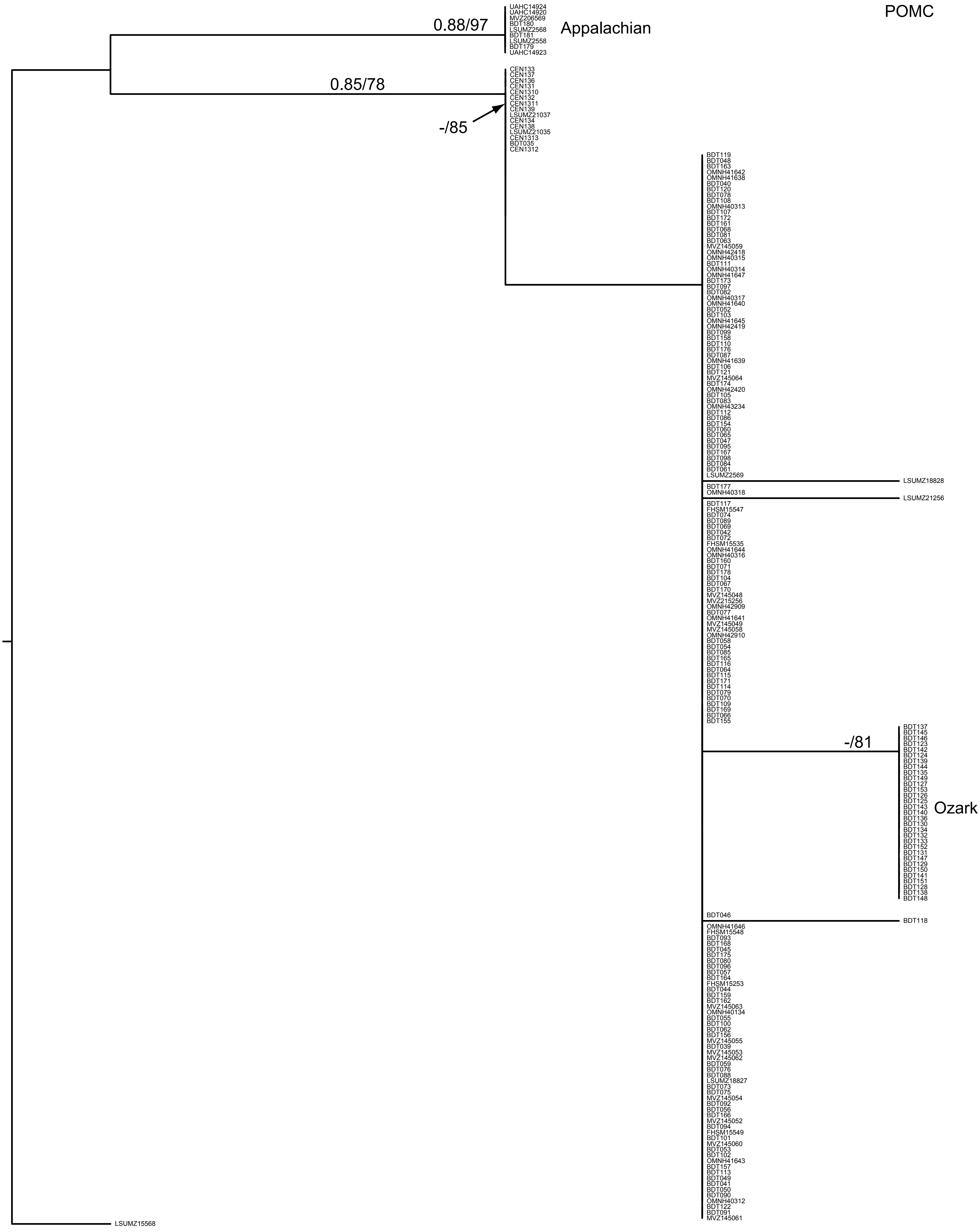
POMC
Appalachian
0.88/97
0.85/78
-/85
-/81
Ozark

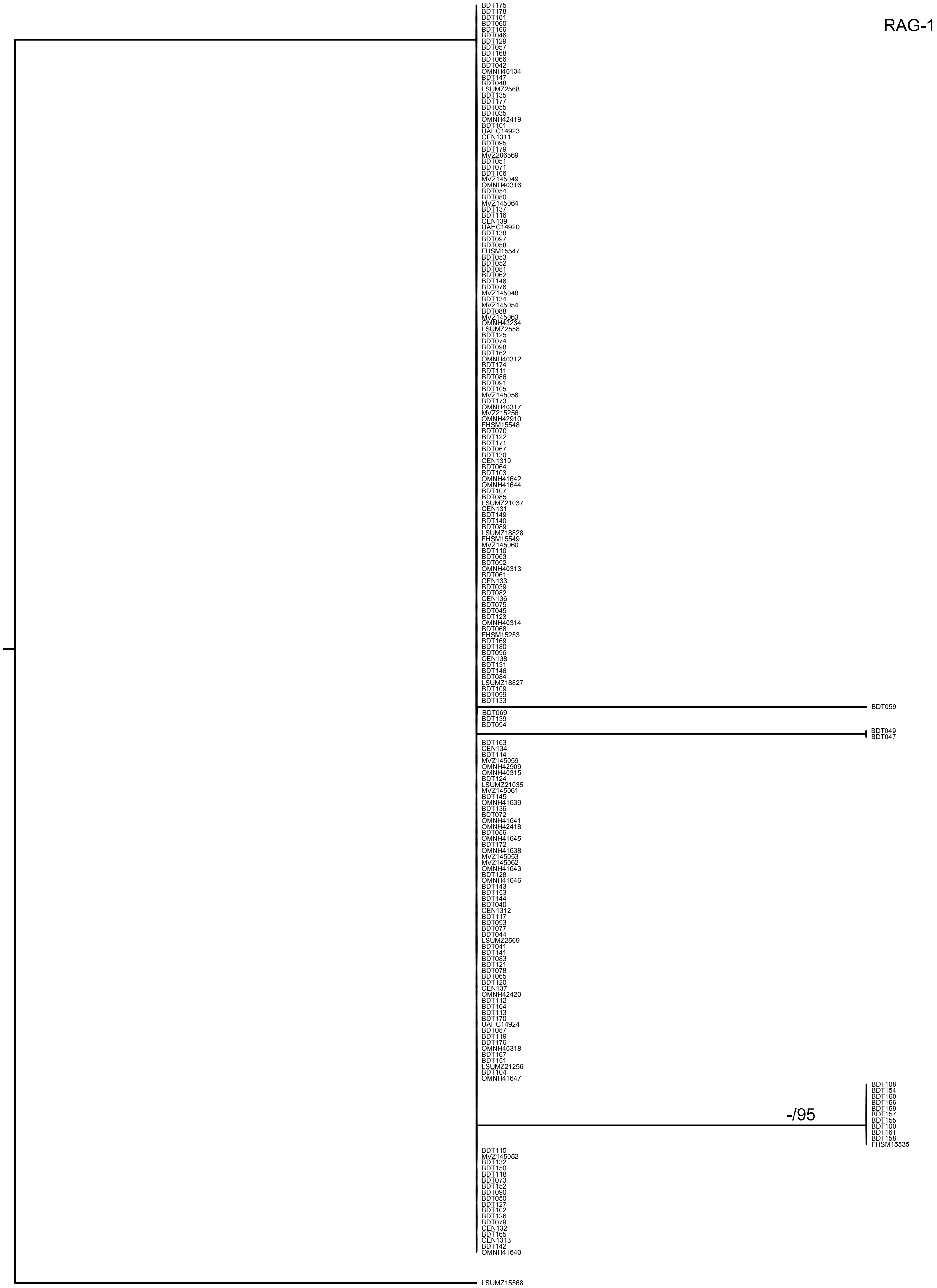
RAG-1
BDT175
BDT178
BDT181
BDT060
BDT166
BDT046
BDT129
BDT057
BDT168
BDT066
BDT042
OMNH40134
BDT147
BDT048
LSUMZ2568
BDT135
BDT177
BDT055
BDT035
OMNH42419
BDT101
UAHC14923
CEN1311
BDT095
BDT179
MVZ206569
BDT051
BDT071
BDT106
MVZ145049
OMNH40316
BDT054
BDT080
MVZ145064
BDT137
BDT116
CEN139
UAHC14920
BDT138
BDT097
BDT058
FHSM15547
BDT053
BDT052
BDT081
BDT062
BDT148
BDT076
MVZ145048
BDT134
MVZ145054
BDT088
MVZ145063
OMNH43234
LSUMZ2558
BDT125
BDT074
BDT098
BDT162
OMNH40312
BDT174
BDT111
BDT086
BDT091
BDT105
MVZ145058
BDT173
OMNH40317
MVZ215256
OMNH42910
FHSM15548
BDT070
BDT122
BDT171
BDT067
BDT130
CEN1310
BDT064
BDT103
OMNH41642
OMNH41644
BDT107
BDT085
LSUMZ21037
CEN131
BDT149
BDT140
BDT089
LSUMZ18828
FHSM15549
MVZ145060
BDT110
BDT063
BDT092
OMNH40313
BDT061
CEN133
BDT039
BDT082
CEN136
BDT075
BDT045
BDT123
OMNH40314
BDT068
FHSM15253
BDT169
BDT180
BDT096
CEN138
BDT131
BDT146
BDT084
LSUMZ18827
BDT109
BDT099
BDT133
BDT059
BDT069
BDT139
BDT094
BDT049
BDT047
BDT163
CEN134
BDT114
MVZ145059
OMNH42909
OMNH40315
BDT124
LSUMZ21035
MVZ145061
BDT145
OMNH41639
BDT136
BDT072
OMNH41641
OMNH42418
BDT056
OMNH41645
BDT172
OMNH41638
MVZ145053
MVZ145062
OMNH41643
BDT128
OMNH41646
BDT143
BDT153
BDT144
BDT040
CEN1312
BDT117
BDT093
BDT077
BDT044
LSUMZ2569
BDT041
BDT141
BDT083
BDT121
BDT078
BDT065
BDT120
CEN137
OMNH42420
BDT112
BDT164
BDT113
BDT170
UAHC14924
BDT087
BDT119
BDT176
OMNH40318
BDT167
BDT151
LSUMZ21256
BDT104
OMNH41647
-/95
BDT108
BDT154
BDT160
BDT156
BDT159
BDT157
BDT155
BDT100
BDT161
BDT158
FHSM15535
BDT115
MVZ145052
BDT132
BDT150
BDT118
BDT073
BDT152
BDT090
BDT050
BDT127
BDT102
BDT126
BDT079
CEN132
BDT165
CEN1313
BDT142
OMNH41640
LSUMZ15568

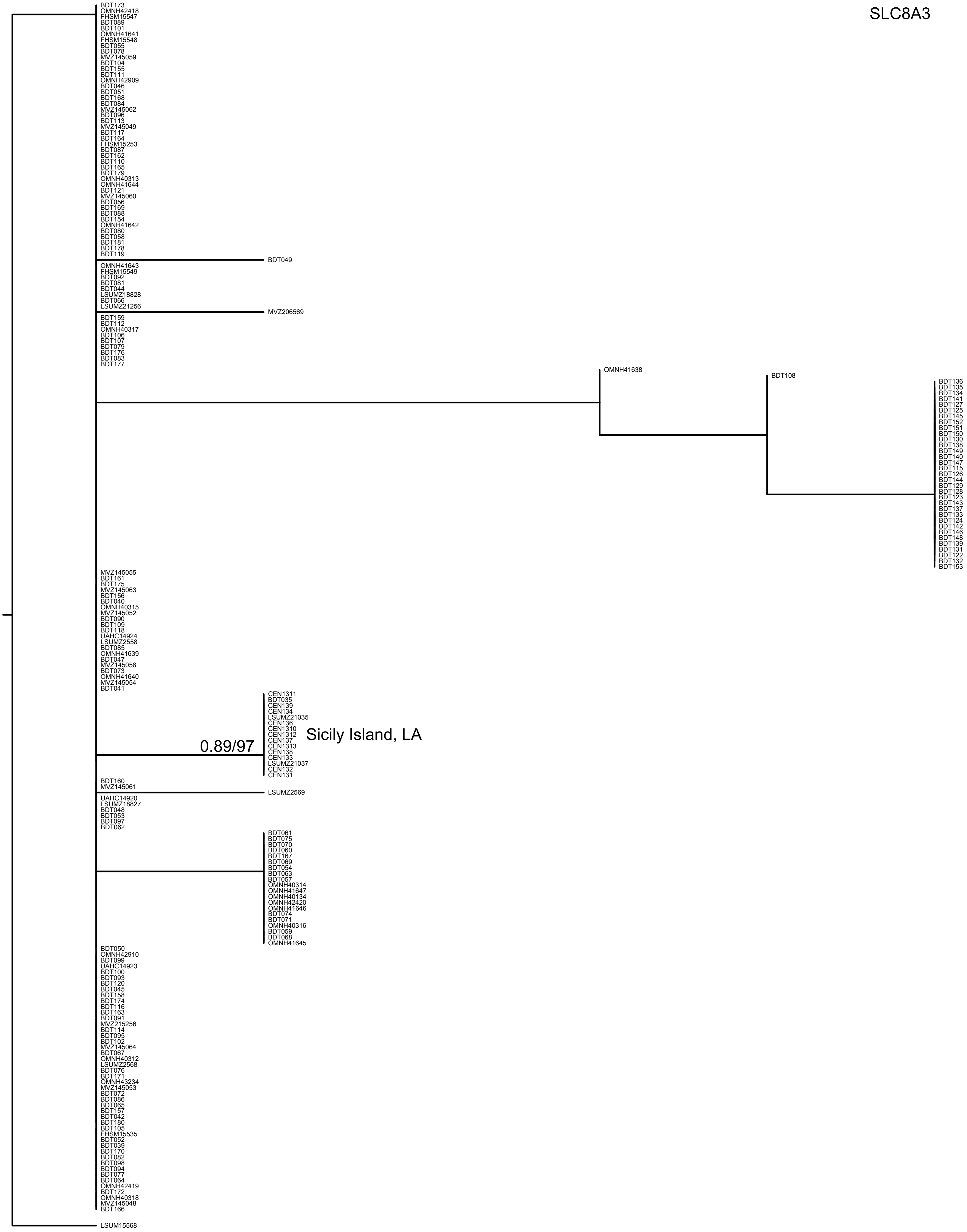

SLC8A3
BDT173
OMNH42418
FHSM15547
BDT089
BDT101
OMNH41641
FHSM15548
BDT055
BDT078
MVZ145059
BDT104
BDT155
BDT111
OMNH42909
BDT046
BDT051
BDT168
BDT084
MVZ145062
BDT096
BDT113
MVZ145049
BDT117
BDT164
FHSM15253
BDT087
BDT162
BDT110
BDT165
BDT179
OMNH40313
OMNH41644
BDT121
MVZ145060
BDT056
BDT169
BDT088
BDT154
OMNH41642
BDT080
BDT058
BDT181
BDT178
BDT119
BDT049
OMNH41643
FHSM15549
BDT092
BDT081
BDT044
LSUMZ18828
BDT066
LSUMZ21256
MVZ206569
BDT159
BDT112
OMNH40317
BDT106
BDT107
BDT079
BDT176
BDT083
BDT177
OMNH41638
BDT108
BDT136
BDT135
BDT134
BDT141
BDT127
BDT125
BDT145
BDT152
BDT151
BDT150
BDT130
BDT138
BDT149
BDT140
BDT147
BDT115
BDT126
BDT144
BDT129
BDT128
BDT123
BDT143
BDT137
BDT133
BDT124
BDT142
BDT146
BDT148
BDT139
BDT131
BDT122
BDT132
BDT153
MVZ145055
BDT161
BDT175
MVZ145063
BDT156
BDT040
OMNH40315
MVZ145052
BDT090
BDT109
BDT118
UAHC14924
LSUMZ2558
BDT085
OMNH41639
BDT047
MVZ145058
BDT073
OMNH41640
MVZ145054
BDT041
CEN1311
BDT035
CEN139
CEN134
LSUMZ21035
CEN136
CEN1310
CEN1312
CEN137
CEN1313
CEN138
CEN133
LSUMZ21037
CEN132
CEN131
0.89/97
Sicily Island, LA
BDT160
MVZ145061
LSUMZ2569
UAHC14920
LSUMZ18827
BDT048
BDT053
BDT097
BDT062
BDT061
BDT075
BDT070
BDT060
BDT167
BDT069
BDT054
BDT063
BDT057
OMNH40314
OMNH41647
OMNH40134
OMNH42420
OMNH41646
BDT074
BDT071
OMNH40316
BDT059
BDT068
OMNH41645
BDT050
OMNH42910
BDT099
UAHC14923
BDT100
BDT093
BDT120
BDT045
BDT158
BDT174
BDT116
BDT163
BDT091
MVZ215256
BDT114
BDT095
BDT102
MVZ145064
BDT067
OMNH40312
LSUMZ2568
BDT076
BDT171
OMNH43234
MVZ145053
BDT072
BDT086
BDT065
BDT157
BDT042
BDT180
BDT105
FHSM15535
BDT052
BDT039
BDT170
BDT082
BDT098
BDT094
BDT077
BDT064
OMNH42419
BDT172
OMNH40318
MVZ145048
BDT166
LSUM15568
